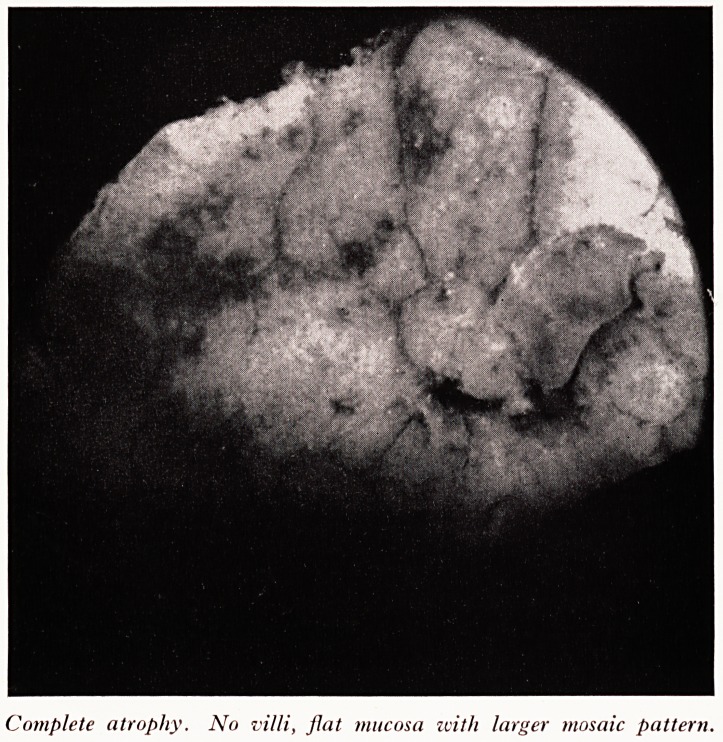# Two Causes for Macrocytic Anaemia in One Patient

**Published:** 1963-01

**Authors:** O. C. Lloyd


### "TWO CAUSES FOR MACROCYTIC ANAEMIA IN ONE PATIENT"

*A Clinico-Pathological Conference held in the University of Bristol on Tuesday, 22nd May, 1962*

CHAIRMAN: DR. O. C. LLOYD

*Dr. O. C. Lloyd:* Our first case was in Southmead Hospital under the care of Dr. Naish, who is at present abroad, and Dr. Mehta has very kindly come to give us the clinical story.

*Dr. S. H. Mehta:* A housewife aged 51 was first seen by Dr. Naish when she was admitted under the care of Dr. Warin for recurrent gravitational ulcers on both her legs in May 1960. By then she had been attending dermatological outpatients for about eight years and had been admitted quite a few times. During her stay in hospital she was referred to Dr. Cates because of macrocytic anaemia and peripheral oedema. Dr. Cates suggested the diagnosis of malabsorption syndrome and thought that all the features in the case were probably due to malabsorption and therefore suggested further investigations.

By that time her haemoglobin was 24 per cent with a mean corpuscular haemoglobin concentration of 32 per cent, and mean corpuscular volume was 121 cubic microns. These are features of macrocytic anaemia and Dr. Lewis kindly did a bone marrow examination which showed predominantly megaloblastic haemopoiesis. The other investigations which were done were a faecal fat excretion, which was 0·83 g in 3 days, which is very minute, and a glucose tolerance curve which was flat, the initial blood sugar being 60 mg per cent, and rising to 106 mg per cent at the end of 2 hours. Maximal histamine test meal showed free acid, serum proteins were 5·9 g/100 ml with slightly raised gamma globulin; serum albumin was normal. Serum potassium, phosphate and alkaline phosphatase were also within normal limits. However the barium meal and follow-through, which was done, showed a grossly abnormal pattern of the distal part of the small gut.

The report on the follow-through is predominantly on the basis of screening, and reads: "the main appearance is of a dilution of barium with a slightly dilated ileum and a little flocculation". According to the radiologist this is probably suggestive of minor malabsorption syndrome.

*Dr. A. E. Read:* What did they think that big blob was?

*Dr. Mehta:* This was the barium in the stomach which is in the left iliac fossa.

At this stage Dr. Cates referred the case to Dr. Naish, specially querying the barium meal and follow-through reports. Dr. Naish suggested that in view of the history of delicate health as a child her malabsorption state might be due to coeliac disease and advised waiting for jejunal biopsy and xylose excretion test. The excretion test was within normal limits i.e. after giving 25 g of xylose the patient excreted 4·8 g within 5 hours. The jejunal biopsy was rather interesting. The X-Ray showed the capsule to be in the left iliac fossa, so Dr. Naish took a biopsy but Dr. Brown reported "normal gastric mucosa"! At this stage, considering the patient's rather unco-operative behaviour, it was decided not to attempt anything further. At the same time we assumed that the stomach was drooping and was right down in the left iliac fossa. From all these tests Dr. Naish considered a diagnosis of macrocytic anaemia due to malabsorption of either folic acid or vitamin $B_{12}$ and decided not to put her on a gluten-free diet. At this stage Dr. Lewis was consulted about treatment, whether to start with vitamin $B_{12}$ or folic acid initially and to assess the reticulocyte response. She was put on vitamin $B_{12}$, 1000 micrograms a day, and the reticulocyte response was observed daily for 7 days. By the end of the seventh day the reticulocyte count was less than 1 per cent. At this stage vitamin $B_{12}$ therapy was discontinued and the patient was put on folic acid 5 mgm three times a day. On the fourth day after starting folic acid therapy the reticulocyte count rose to 10 per cent; it was then thought that this was probably a case of folic acid malabsorption causing macrocytic anaemia. She was then transfused with packed cells because the Hb was only 24 per cent. Her Hb rose from 24 per cent to 74 per cent, and in under ten days her blood picture became normocytic. She made a fairly good recovery and was discharged home on maintenance folic acid treatment which was 5 mgm t.d.s. From then onwards in outpatients a careful watch was kept on her condition and Hb, and she remained fairly well until November 1961 when she was readmitted as an emergency having complained of severe breathlessness, generalised oedema, tiredness and chest pains for one month. For the two weeks before admission she was almost all the time in bed and was almost orthopnoeic. On examination she was very pale and showed gross heart failure, B.P. was 180/80, pulse 120/min, regular. Her tongue at this time was quite normal. From the G.P.'s letter I gathered she was on folic acid and he had also put her on Imferon thinking that she might have iron deficiency anaemia this time. Hard, irregular slightly tender lumps were palpated in both breasts, and her liver was palpable and tender but was not suggestive of any secondary malignant condition. No other masses were felt. The Hb was 34 per cent and R.B.C.'s were mainly orthochromic; the blood film was suggestive of leucoerythroblastic anaemia, about 78 nucleated R.B.C.'s being seen per 100 W.B.C. There was also evidence of thrombocytopenia. Again Dr. Lewis was consulted and he very kindly did a bone marrow examination (on a Sunday morning) which showed cells of anaplastic nature that were not producing mucus. It was decided that this was obviously a case of leucoerythroblastic anaemia due to malignant bone marrow metastases, most probably from cancer of the breast. She was transfused again with packed cells and she improved a little for about 2 to 3 weeks and then gradually deteriorated for another 2 to 3 weeks and finally died of bronchopneumonia.

So, summarizing the case we have here a patient who had two distinct diseases, the first one was macrocytic anaemia, due to folic acid malabsorption, from which she made a recovery on maintenance treatment. Later on she developed a fatal illness with presentation of leucoerythroblastic anaemia from a common cause, carcinoma of the breast with bony metastases.

*Dr. Lloyd:* When she was in hospital in May 1960 were her breasts examined, and were they completely free from tumour?

*Dr. Mehta:* I gather from the notes the breasts were examined and no abnormality was found.

*Dr. A. B. Raper:* Can you remind us, please, which month she was sent home, and were there any readings of Hb subsequently?

*Dr. Mehta:* She was sent home on 4th July and when she was seen in outpatients on 3rd August 1960 her Hb was 96 per cent, M.C.H.C. 34 per cent. Then on 21st September 1960 she was seen by Dr. Naish; fairly well but again had ulcers on her legs and oedema. She came in at that time under Dr. Warin. On admission her Hb was 96 per cent, but it fell below that when she was under his care. Then she was seen in December 1960 with her leg ulcers healed. She also had a widespread inconstant back pain and was incontinent. I saw her on 27th March 1961. She had had a fall a month before, injuring her back. Since then she had not been able to walk. She was complaining of pain in the back. An X-ray of sacro-iliac joints and lumbar spine was reported as "no evidence of abnormality in bones seen. Abnormal bilateral suprarenal calcification seen".

*Dr. Lloyd:* To what did you attribute the pain in her back?

*Dr. Mehta:* I thought that after the fall she had probably hurt her back, but there was no evidence of fracture. I thought it could be either muscular or fibrositis. There was also suspicion that she might have some sort of metastases.

*Dr. Lloyd:* Even then?

*Dr. Mehta:* Even then, because of constant continuous back pain.

*Dr. Lloyd:* To what did you attribute the calcified shadows seen on the X-ray of her abdomen?

*Dr. Mehta:* The radiologist has mentioned very carefully that calcification is seen in the region of the suprarenals, just about equal on each side. The shadow is oblique and it is difficult to think that it is anything else than the suprarenals. She had a history of tuberculous pleurisy in her "teens".

*Dr. Read:* Was it seen on the previous barium meal?

*Dr. Mehta:* No.

*Dr. Lloyd:* Would Dr. Lewis like to describe his haematological findings in this case?

*Dr. F. Lewis:* When we first saw her she had a typical megaloblastic marrow with a clinical diagnosis of malabsorption. There was free acid present suggesting a folic acid sensitive anaemia, but we were not then doing folic acid or vitamin $B_{12}$ levels. One way however, that you can investigate this is to do what is called "the double reticulocyte response". We started the patient on vitamin $B_{12}$ doing daily reticulocyte counts; then 3 days later, we added folic acid in order to get a possible 7-day reticulocyte peak from each: there was no 7-day peak from the vitamin $B_{12}$ but there was a 7-day folic acid peak of 10 per cent reticulocytes; the haemoglobin rose, I think, to 54 per cent and then she was transfused.

On the second occasion: the finding of these tumour cells. I am very grateful to hear that I came in on the Sunday. In fact, I had a very keen and enthusiastic registrar and it was he who came in on Sunday. He did the sternal marrow puncture because the patient had been sent in by the general practitioner with a leucoerythroblastic anaemia and with such an anaemia you suspect secondary carcinoma. With great difficulty he obtained only a dilute specimen with hardly any cells in it; but something we have learnt by experience is that you must spin down the deposit and patiently do histology on the bits even if the bits themselves are microscopic. This we did and there was an anaplastic carcinoma. We were even able to do a P.A.S. stain to see whether it was mucin-producing. This we thought was negative, but the appearance suggested breast as a possible primary site. And that is all I have to tell you.

*Dr. Lloyd:* I have asked Dr. Read to come and collect together all the clinical threads in this case, and tell us what he thinks about it.

*Dr. Read:* This illness seems to fall into two phases as we have seen. I think there is some evidence here of malabsorption of the "mucosal defect" type. The reason I say this is that she has most of the abnormalities one finds in a proximal bowel lesion, that is she has a flat glucose tolerance curve; she is anaemic and this is almost certainly a folic acid deficiency, folic acid being widely absorbed up and down the alimentary tract one needs a very diffuse mucosal deficiency in order to get anaemia due to malabsorption of it. The only type of malabsorption that will make you folic acid deficient is a mucosal lesion of the idiopathic steatorrhoea or coeliac disease group. I must admit however that the normal xylose excretion is a blow.

You might say that we have already shown that she had not got steatorrhoea so how can we say that she has this mucosal lesion? Well, admittedly it is sometimes very difficult to show up steatorrhoea in patients who are in hospital. You know very well that patients who have diarrhoea when they come into hospital almost always celebrate the event by having tenacious constipation and it is very difficult then to get satisfactory fat balance studies on them. This is why I think that fat balance studies should be done when patients are up and about the ward and on as high a fat intake as is possible. The fat balance here was negative but I do not think this excludes there being a mucosal abnormality, or even latent steatorrhoea.

The next point is the biopsy. Owing to this very peculiar position of the stomach Dr. Naish's colleagues were of course unfortunate and did not get the capsule through into the small gut at all so they could not get a biopsy. One small point does come up

PLATE I

*Normal jejunal mucosa. Note the villi as separate finger-like processes.*

PLATE II

*Subtotal villous atrophy; mucosa convoluted but without distinct villi.*

PLATE III

*Complete atrophy. Mucoas flat, with a fine mosaic pattern; no villi.*

PLATE IV

*Complete atrophy. No villi, flat mucosa with larger mosaic pattern.*

in this. It is very wise to outline the polythene tubing when you are doing this sort of biopsy and that would have told them before they fired the capsule that it was still in the stomach.

*Dr. Read now showed photographs of intestinal mucosa taken through a dissecting microscope showing:*

1. *Normal mucosa (Plate I).*
2. *Mucosa from a patient with only mild steatorrhoea but with folic acid malabsorption. The villi were convoluted rather like cerebral convolutions and did not stand up, separated, as in the normal example. A histological section showed that the villi were fatter than normal and distorted so that they were not true "villi" at all but convolutions of the mucous membrane. This is known as subtotal villous atrophy and would be the sort of lesion Dr. Read would have expected to find in the case under discussion (Plate II).*
3. *Mucosa from a patient with gross steatorrhoea. The mucous membrane was flat with no villi or convolutions. The histological section showed complete villous atrophy. This is the lesion seen in coeliac disease. (Plates III and IV.)*

I think it is possible that this lady had a mucosal defect and that it might have shown up if we had done a biopsy in the right place, but that we should not be put off just because she had not got steatorrhoea. Now it is significant that she responded well to folic acid, her Hb went up and something else seems to have happened at this point. Dr. Lewis has shown us that this was carcinomatosis. Whether it is from the breast or whether it is from somewhere else it is difficult for the clinicians to say. There were two abnormal breasts. I should have thought this was a little unusual, but one could get a growth in both. I just wonder whether it is possible to get secondary deposits in both breasts from somewhere else, but there certainly is a neoplastic lesion. The way we can connect these two, i.e. malabsorption and neoplasia, is very difficult. Now people have described neoplastic change occurring in idiopathic steatorrhoea. Some of them have been carcinomas, more commonly they are reticuloses. Dr. Gough and Dr. Naish and I have described five patients with this combination. This would have been a nice way of drawing the threads together but I am afraid it is not going to work out (*a*) because it is a carcinoma and not a reticulosis and (*b*) because if it were a carcinoma I would have expected it to be mucus-secreting if it came from the bowel. So I would say that this lady had malabsorption, that she then developed carcinoma probably of the breast or breasts and that she died with widespread malignant deposits in the bones causing the bone pain.

The thing that does not fall into place, and I do not know what the answer is, is this funny calcification which does not seem to have been there a year before. It does not look like pancreatic calcification, I wonder if it could be calcification in secondaries in the liver, which occasionally occurs?

In summary I would say this lady probably had a malabsorption syndrome, that her mucosa might show an abnormality, if it was still intact; that she responded well to folic acid given for this and that she did not have steatorrhoea. She developed a malignant disease which spread to her bone marrow causing a second type of anaemia, a leuco-erythroblastic one, and the most likely site of this, in view of the fact that we are told there was swelling in the breasts, is the breast. I would have liked to have tied it a little closer together and said it came from the gut, but I am afraid that would be wrong.

*Dr. Lloyd:* Thank you very much. I take it then that you agree with Dr. Mehta that she has got two separate diseases, you would like to be able to link them together but you are reluctant to do so quite definitely, and you say that you would rather expect if the growth had been from the intestine that it would have been mucus-secreting. I think I have only seen one of your cases of intestinal neoplasm that came from a case of malabsorption syndrome and my recollection is that it was a reticulosarcoma. That of course would not be mucus-secreting. I am not quite sure what your other ones were.

*Dr. Read:* We have not seen any, but a couple of small bowel carcinomata have been reported. Presumably they would be mucus-secreting.

*Professor A. V. Neale:* Could you tell us something about the content of folic acid in diets? Is there any reason to suppose that it might be dietary deficiency rather than malabsorption?

*Dr. Read:* This is a thing we are interested in at the moment. Nobody knows how much folic acid one needs normally. Most people reckon that about 200 micrograms is the average daily intake. Of course about half of that is destroyed by cooking so most people actually get about 100. Now this is perfectly adequate to keep one from becoming folic acid deficient but we have been interested in a group of patients with very peculiar ideas as to what they should eat and living mainly on bread and butter and cups of tea fortified with lots of sugar, which keeps up their calory intake and we have noted folic acid deficiency occurring in them. It is pure folic acid deficiency because these patients respond to very small doses of folic acid, that is anything in the order of 100–200 micrograms/day. The patient with vitamin $B_{12}$ deficiency of course will respond to a big dose of folic acid, but a patient with pure folic acid deficiency will respond to very small doses of folic acid and one can therefore prove in this way whether a patient is folic acid deficient or not. Of course their serum folic acid levels are very low, their FIGLU's are positive, their intestinal villi are normal and they have no steatorrhoea. There is no evidence of malabsorption syndrome, so we think that megaloblastic folic acid deficiency due to dietary deficiency is fairly common particularly in rather eccentric middle aged women either living alone or having very strange ideas about their diet. Dr. Gough and I and others have been chasing round Bristol calling on eccentric middle-aged ladies and trying to prove this point, which I think is a genuine one!

Could this lady have it? Well I do not see why we should postulate this because it seems likely that there must be a mucosal abnormality if she had a flat glucose tolerance curve.

*Professor Neale:* Would you go as far as that: she *must* because she has a flat glucose curve? Did you put the glucose in the duodenum?

*Dr. Read:* No. I didn't.

*Professor Neale:* It may not move out of the stomach.

*Dr. Read:* I think it did because the serum glucose went up 40 mgs per cent and you must have had something to do this.

*Professor Neale:* I have seen flat glucose tolerance curves in all sorts of circumstances and I would regard it as full of fallacies.

*Dr. Lloyd:* Professor Neale, could you please tell us why it is necessary to put it into the duodenum rather than into the stomach?

*Professor Neale:* Because you are sure it is in the right place for absorption.

*Dr. Lloyd:* Does it not go there eventually?

*Professor Neale:* It may not.

*Dr. Lloyd:* What happens to it?

*Professor Neale:* It stays longer in the stomach.

*Dr. Lloyd:* And then?

*Professor Neale:* It passes on slowly. Too slowly. It is the time factor, isn't it?

*Dr. Read:* Well, I would have said that the glucose tolerance curve is a reasonable method of detecting whether the upper small bowel is normal or abnormal.

*Professor Neale:* One cannot argue that a flat curve is not fallacious. It is a rise that is a positive normal curve. You can argue from that. However I won't prolong the argument. But may I ask one question: what dose of folic acid was given in the therapy to produce 10 per cent reticulocytes? What do you mean by "a small dose"?

*Dr. Read:* 100 micrograms.

(Inaudible question.)

*Dr. Read:* That brings up a very important point. It is possible, I think Dr. Raper and Dr. Lewis will agree with me here, that if you have a tumour that is growing rapidly, such as a foetus, or a carcinoma or a sarcoma or reticulosis, it is possible to get a conditioned folic acid deficiency. Folic acid is necessary for the production of nucleic acid, all cells need it and so a deficiency is common in pregnancy, particularly if you have two tumours, i.e. twins, that is right, is it not Professor Neale, it is much more common in twin pregnancies?

*Professor Neale:* More so in triplets!

*Dr. Read:* And it is well recognized in reticulosis or in haemolytic anaemia that patients will develop a secondary folic acid deficiency. Whether one could tie this case up another way and say this lady had a tumour, a widely disseminated one, which was producing a condition of folic acid deficiency or not I would not like to say. I would be content with one explanation of folic acid deficiency associated with malabsorption, and I would say that this is the most likely cause because she did remain in reasonable health, I think, for a year in between these two illnesses whereas I think if the previous secondary cause had been operative at the beginning she would not have survived as long.

I do not think it is a feature of carcinomata so much as reticulosis. Perhaps Dr. Raper or Dr. Lewis would say something?

*Professor Neale:* Was the xylose curve normal or abnormal?

*Dr. Read:* The blood xylose excretion—we do not plot blood levels—was normal, I am afraid.

*Dr. Lloyd:* I think the time has come for us to ask Dr. Draisey for the post mortem account and to tell us what he really found.

*Dr. T. F. Draisey:* Because of the problem of malabsorption here, this post-mortem was done in two stages. Very shortly after death we obtained a specimen of the small bowel and gave it to Dr. Brown who reported that there was minimal blunting of the villi but no inflammatory exudate in the lamina propria of the jejunum. That was half an hour after death.

Then more at leisure the post-mortem proper was carried out. She was a well nourished middle-aged woman with oedema of both legs, ulcers and eczema over each internal malleolus. Both breasts felt lumpy on palpation. On examination there was no discrete tumour, rather diffuse greyish-white tissue in the breast, rather like one finds in fibro-adenosis. So I took sections from each breast. The adrenals were the calcified organs—both were calcified with recognizable adrenal tissue on each side. The spleen weighed 666 g, with a firm cut surface and with loss of medullary pattern. The liver weighed 1931 g and was rather pale with a few discrete white areas.

The liver was not very enlarged, but the spleen was large and flattened, it was rather evenly brown throughout. The cut surface of sternum and ribs were an even grey colour but with discrete white areas of metastatic tumour.

The intestines appeared normal macroscopically and apart from that there was nothing else of note.

Section from the left axillary lymph node shows moderately well differentiated adenocarcinoma of the breast. Section of the breast shows a rather less well developed tumour, rather anaplastic diffuse carcinoma.

Section of the sternum shows replacement of the marrow by carcinoma, rather small cells with quite an amount of scirrhous reaction round them. There is no recognizable field of haemopoietic tissue whatsoever.

Microscopically the liver shows secondary tumour, much more like carcinoma of the breast than the actual primary! A section from the spleen bears out the blood-borne nature of the metastases, by showing secondary carcinoma. Also in the splenic tissue one sees rather a large number of very small dark-staining cells in areas away from the secondary tumour. They seem to be nucleated red cells. Although I can find no megakaryocytes there is a considerable amount of haemopoiesis going on in the spleen.

So the conclusion here is that this was a carcinoma of the breast with an unobtrusive primary, diffuse bony secondaries and extra-medullary haemopoiesis in the spleen. This was present both in the liver and the kidney too. As to the malabsorption, well if there was malabsorption it was of a very minimal character, as borne out by the histology.

*Dr. Lloyd:* The carcinoma that you demonstrated to us came from the left breast. Did you find an identical carcinoma in the right breast?

*Dr. Draisey:* No, in the right breast I found fibro-adenosis and lobular hyperplasia only—no cancer at all.

*Professor Neale:* Would you tell us more about this "unobtrusive primary carcinoma"? Would it be clinically recognizable?

*Dr. Draisey:* It would not be recognizable to me.

*Dr. Lloyd:* We did enquire about that when Dr. Mehta was giving us the clinical findings. Apparently in May 1960 when she first came in they did examine her breasts and there was no tumour there. I do not know whether Mr. McPherson would like to tell us about the difficulties of palpating tumours in breasts?

*Mr. A. G. McPherson:* If a patient complains of symptoms referable to the breast and a careful examination is made with the express purpose of detecting a lesion, it is possible to detect quite small tumours. But this patient had no such symptoms and I think that the ordinary routine examination which would have been carried out, even if this had been done by a surgeon, might well have failed to reveal a small lesion. So the absence of a small tumour 2 years ago cannot be assumed.

*Dr. K. R. Gough:* Going back to the possible malabsorption state I would like to enquire whether this lady was taking barbiturates? It is possible that if she had been taking barbiturates in excessive doses allied to a diet deficient in folic acid that this could have caused her megaloblastic anaemia and in fact she may not have had an actual mucosal abnormality of the small bowel.

*Dr. Mehta:* There is no record of this.

*Professor Neale:* Does the leucoerythroblastic anaemia in this type of case arise gradually or does it come on insidiously or does the blood picture sometimes change very rapidly?

*Dr. Lewis:* It is a very difficult question to answer. You can have a limited erythroblastosis with considerable change in the bone marrow and you can have a considerable erythroblastosis that is not reflected in the marrow.

The megaloblasts in the first marrow examination were very definite megaloblasts—consistent with malabsorption.

*Professor Neale:* Well of course under the influence of malignancy all sorts of metabolic processes are deranged aren't they? I do not think there is any point in fixing it down to A or B. There is a lot of disorder in the body in malignant disease and it interferes with all sorts of enyzme activities.

*Dr. Read:* Were you suggesting, Professor, that the original megaloblastic anaemia was conditioned by secondaries in the bone marrow, at the onset of the .......

*Professor Neale:* No, I don't think so. I still think we have not discovered exactly why she developed megaloblastic anaemia except there was a strong suspicion of folic acid deficiency. What foods are rich in folic acid?

*Dr. Read:* Green vegetables, fruit and liver.

*Professor Neale:* It is a very interesting point that parsley contains more folic acid than any other common form of vegetable. A clinician whom I knew many years ago used to put patients who had "anaemia" on large amounts of parsley sauce. Years later it was discovered that it contained folic acid 60 micrograms/100 g.

*Dr. Read:* I think I must defend this business about malabsorption. I do not think you have shown that there is not malabsorption. The histological report on the mucosa says there is "minimal blunting". I mean you couldn't have said it was more normal than that without saying there is nothing, but I hope I have shown that you can't just by taking a slice of tissue and looking at so called villi pronounce a verdict of normality or otherwise.

*Dr. Lloyd:* Would Dr. Brown like to defend his reported opinion about this minimal blunting?

*Dr. N. J. Brown:* This was the sort of mucosa where I was not prepared to say it was absolutely normal but it was not one that was very definitely abnormal. I know that is saying the same thing as "there was minimal blunting" but I do not think my opinion of the section helps to solve the problem one way or the other.

*Dr. M. E. H. Halford:* It may be of some interest for the future to know that we have looked at *post mortem* specimens under the dissecting microscope and since the effect of *post mortem* autolysis is only to remove the surface epithelium it does not change the dissecting microscope view of the changes, in fact it is probably better with *post mortem* specimens to use the dissecting microscope than to examine an ordinary section; one sees the changes which are present in your biopsies in exactly the same way.

*Dr. Brown:* Perhaps I could read you my exact report. It says: "Piece of small intestine 9 × 3 cm, there is blunting and broadening of some of the villi, suggestive of a mild degree of villous atrophy. There is no inflammatory infiltration of the lamina propria."

*Dr. Read:* You did use the dreaded words "villous atrophy" then?

*Dr. Brown:* "Suggestive of a mild degree."

*Dr. Lloyd:* I think we might leave the small intestine, just for a moment. Dr. Raper, I wonder if you would like to try to help us decide about this anaemia, whether the first one she was suffering from in May 1960 could have been due to the secondary carcinoma that developed or whether it is really two separate things that have been happening.

*Dr. Raper:* I do not think that I can decide that, but I can at least quote two things that I think are of interest. If you accept the fact that the reticulocyte response was one to the folic acid and not to vitamin $B_{12}$, as we well may, then we could rule out any diversion of the folic acid to a tumour which was inapparent at that time and only caused any change in the blood pattern as far as we know within a month or two before the patient died. I would like to ask what was the blood group of this lady? And does Dr. Read know of any association between any blood group and malabsorption as there is between Group A and cancer?

*Dr. Lewis* Her blood group was A Rh positive.

*Dr. Read:* I do not know of any association between blood groups and malabsorption. I think it has been worked out but I think it has been shown that there is not a close correlation like there is with salivary tumours and pernicious anaemia, peptic ulcer etc. I think the answer is "no", there is no association with blood groups. If anyone does know the correct answer perhaps he would tell us?

*Dr. Raper:* I wonder if anyone can tell us whether giving this lady folic acid encouraged her tumour to grow?

*Dr. Lloyd:* To conclude, then, this was the case of a woman who first had a megaloblastic anaemia, which was believed to be due to defective folic acid absorption, and who later developed a leucoerythroblastic anaemia due to secondary carcinoma of the marrow. The general opinion is that they were two separate conditions, but the probability cannot be ruled out that she may have had secondary carcinoma of the marrow quite early on.

## Figures and Tables

**Figure f1:**
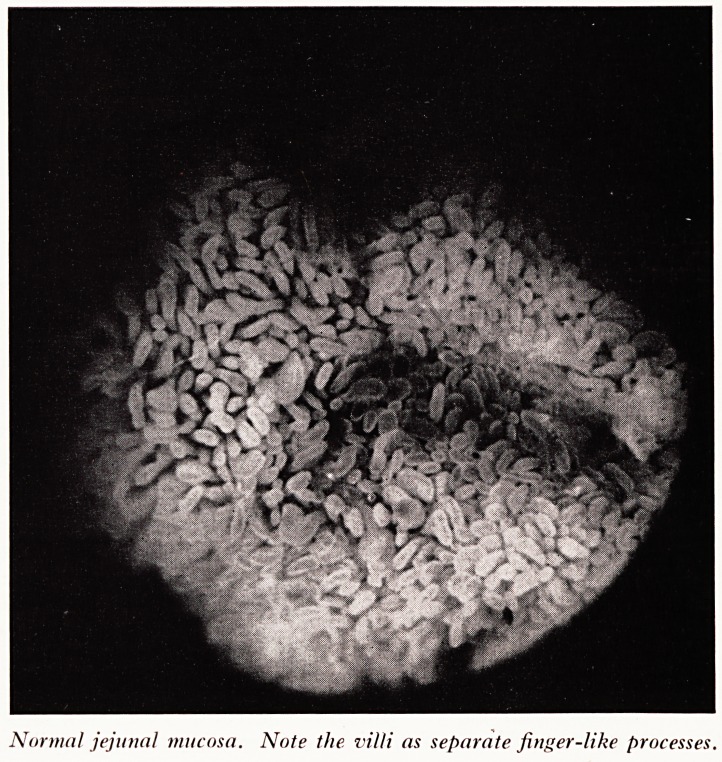


**Figure f2:**
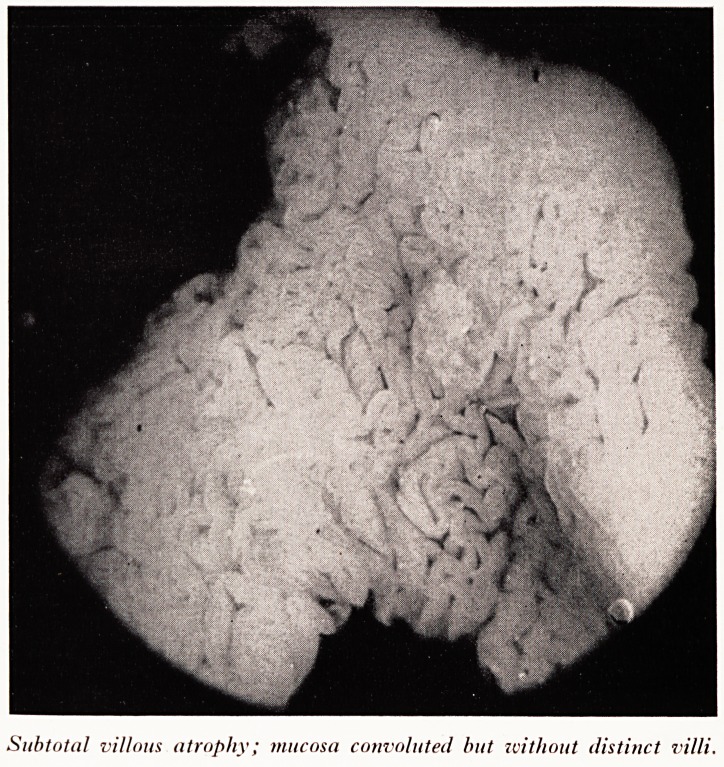


**Figure f3:**
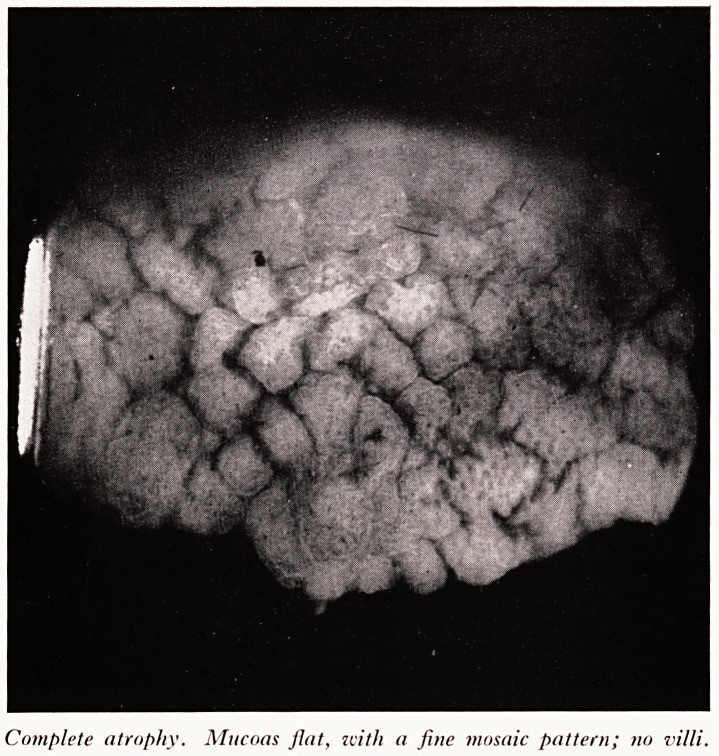


**Figure f4:**